# Functional Morphology of the Pedal Musculature and Grip Force Estimation in the Red‑and‑Green Macaw (*Ara chloropterus* Gray, 1859)

**DOI:** 10.1002/jmor.70152

**Published:** 2026-07-29

**Authors:** Sérgio Roberto Posso, Vinicius Camargo Tavares, Ana Caroline M. Barbosa

**Affiliations:** ^1^ LESCAN – Laboratory of Ecology, Systematics and Conservation of Neotropical Birds Mato Grosso do Sul Federal University Três Lagoas MS Brazil

**Keywords:** biomechanics, Feet, myology, Psittaciformes

## Abstract

Psittaciformes possess zygodactyl feet that support perching, climbing, inverted postures, and foot‐mediated food manipulation. Despite the functional importance of these behaviors, the myological and biomechanical bases of digital performance remain poorly documented in large‐bodied psittacids, particularly macaws. Here, we investigate the digitized musculature and estimate the functional force output of the foot in the red‐and‐green macaw. We describe the intrinsic and extrinsic muscles acting on the digits, quantify muscle length and mass, and estimate digit‐specific pinching forces based on physiological cross‐sectional area and mechanical advantage. Digit IV is the longest digit, and its primary flexor, *m. flexor perforatus digiti IV*, is the longest and most massive digital flexor. Consistent with this morphology, digit IV generated the greatest pinching force (8.63 ± 1.52 N), indicating a dominant role in force production. The remaining digits are shorter and associated with more slender musculature, resulting in lower force outputs (digit II: 4.51 ± 0.92 N; digit III: 2.29 ± 0.30 N; digit I [hallux]: 1.06 ± 0.15 N). The combined force of the posterior digits (I + IV; 9.69 ± 1.74 N) approximates that of the anterior digits (II + III; 6.80 ± 1.22 N), indicating a near‐balanced anterior–posterior force distribution that likely enhances stability during perching and climbing while facilitating precise manipulative behaviors. Apparently, the total pedal force (16.49 ± 3.22 N) scales with body size in Psittaciformes; however, this hypothesis requires testing by using explicit allometric and phylogenetic comparative approaches. Finally, this foot‐gripping force is lower than that reported for large raptors (Accipitriformes and Falconiformes), reflecting functional specialization of the macaw pes for controlled arboreal locomotion, inverted postures, and fine motor manipulation rather than maximal force generation.

## Graphical abstract

Integration of detailed hindlimb anatomy with biomechanical analyses demonstrates that pedal performance in the red‐and‐green macaw is driven by specialized muscle architecture and force distribution. Hypertrophy of digit IV flexor musculature, together with modifications of the intrinsic and extensor muscles, enhances grasping stability, climbing, and food manipulation rather than maximal force generation.

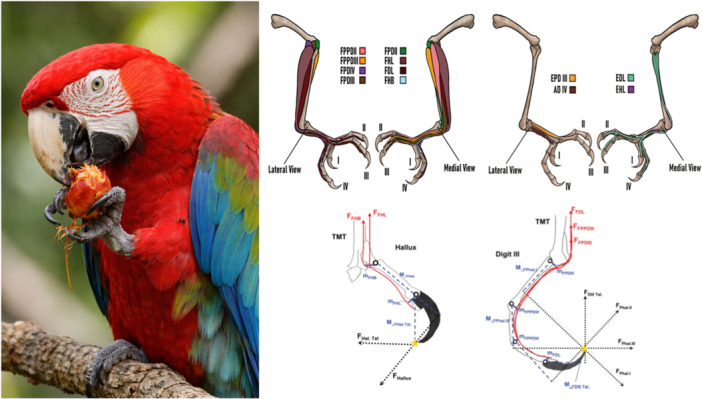

## 1 Introduction

Grasping with an appendage is a fundamental functional capacity with important evolutionary implications, underpinning the development of fine motor control and precision prehension in many vertebrate lineages (Manzano et al. 2008; Abdala et al. 2009). Despite its central role in locomotion, feeding, and reproduction, prehensile behavior has received comparatively limited attention (Sustaita et al. 2013). In the pes, grasping involves coordinated positioning of the digits and appropriate limb displacement in space, and can be defined according to the degree of precision required for object acquisition and retention (Mackenzie and Iberall 1994). Comparative studies of tetrapod limb musculature have revealed extensive homologies underlying these movements, suggesting that prehensile behavior may be phylogenetically conserved, potentially even more so than the pentadactyl limb pattern itself (Abdala and Diogo 2010; Kardong 2011), although this hypothesis remains insufficiently tested due to limited data (Sustaita et al. 2013).

In birds, the forelimbs are highly specialized for flight, and as a consequence, many complex and fine motor functions have been transferred to the beak and hindlimbs (Proctor and Lynch 1993; Sustaita et al. 2013). Avian pedal grasping has traditionally been associated with arboreal locomotion, with emphasis on digit arrangement and musculoskeletal organization as mechanisms for balancing internal and external forces and maintaining substrate attachment (Bock and Miller 1959; Backus et al. 2015). An important precursor to the evolution of prehension in birds was the inversion and functional establishment of the posterior digit (hallux) as an opposable element (Feduccia 1999; Middleton 2001). Although high levels of pedal prehensility are commonly associated with arboreal locomotion in birds, feeding behaviors have also been implicated as important selective pressures (Sustaita 2008; Fowler et al. 2011). In fact, despite early selection for perching, avian pedal morphology has been shaped by multiple, and sometimes conflicting, functional demands related to locomotion and feeding, resulting in a remarkable diversity of foot forms and functions across birds (Bock and Miller 1959).

Prehensile function in feeding contexts—defined as the use of the feet to manipulate food items—is less widespread but phylogenetically broad, and it has been interpreted as expanding dietary breadth by enabling the handling of large or resistant food items (Clark 1973; Sustaita et al. 2013). This functional shift is especially pronounced in Psittaciformes, with greater reliance on coordinated foot–beak interactions (Brown and Magat 2011). These birds are readily recognized by their remarkable pedal dexterity and manipulative abilities (Forshaw 1989; Collar 1997), as well as by their curved, robust beaks and highly pseudoprokinetic cranial articulation (Zusi 1993; Tokita 2003), especially in macaws (Porto 2004; Faillace et al. 2025; Posso et al. 2026). All Psittaciformes species possess zygodactyl feet, with digits II and III oriented anteriorly and digits I and IV oriented posteriorly (Carril et al. 2024). This digital configuration enables effective perching, as well as climbing, hanging, and terrestrial locomotion (Bock and Miller 1959; Roderick et al. 2019; Dickinson et al. 2022). Parrots frequently use the hindlimbs to grasp and manipulate food, bringing it to the beak while maintaining postural stability, and they are capable of anchoring themselves to branches to assume characteristic inverted or descending postures during foraging (Forshaw 1989; Collar 1997).

These behaviors, together with the mobility and mechanical robustness of the jaws, allow parrots to exploit hard fruits and seeds or otherwise inaccessible food resources, thereby reducing interspecific competition (Backus et al. 2015; Collar 1997). The execution of such gross and fine motor tasks requires precise integration of skeletal elements, ligaments, and musculature. In this way, pronounced development of the tarsal musculature and digital tendons has been associated with enhanced pedal performance in several psittacine taxa (Carril et al. 2014; Backus et al. 2015; Roderick et al. 2019; Dickinson et al. 2022). Thus, given the degree of dexterity required for climbing, inverted postures, and foot‐mediated food manipulation, tarsal musculature and digital tendons of psittacines are expected to exhibit functional specializations distinct from those observed in other avian groups (Berman 1984; Carril et al. 2014).

Studies of avian anatomy intensified during the late twentieth and early 21st centuries, largely aimed at generating morphological characters for taxonomic and phylogenetic analyses (Livezey and Zusi 2001). Within Psittaciformes, relatively few investigations have addressed the functional morphology and evolutionary significance of the musculoskeletal system, and such studies remain particularly scarce for this order (Sustaita et al. 2013; Carril et al. 2014; Dickinson et al. 2022; Faillace et al. 2025). This gap is especially evident in species‐specific studies of macaws that integrate detailed anatomical descriptions with biomechanical modeling approaches (Posso et al. 2026). In fact, functional interpretations of psittacine locomotor anatomy remain limited, being restricted primarily to the studies of Carril et al. (2014), Roderick et al. (2019), and Dickinson et al. (2022). To date, no study has integrated detailed myological descriptions with gripping force estimation within a single macaw species.

The Red‐and‐green Macaw (*Ara chloropterus*) provides an excellent model for such an analysis. This large macaw reaches approximately 90–95 cm in total length and weighs between 1250 and 1700 g (Sick 2001; Guedes 2012). It is widely distributed throughout Central and South America (Collar 1997). Although currently classified as Least Concern by the IUCN (2026), many populations are experiencing declines as a result of continued habitat loss and pressure from the pet trade (Collar 1997; Olah et al. 2016). The species is a dietary generalist, dominated by large seeds (Matuzak et al. 2008; Scherer‐Neto and Terto 2011; de la Parra‐Martínez et al. 2019; Lee et al. 2014; Ragusa‐Netto 2022, 2024). Although the cranial osteology, jaw musculature, and bite force of the red‐and‐green macaw have been described within comparative frameworks (Porto 2004; Harrison et al. 2024; Faillace et al. 2025), the hindlimb—particularly the digital musculoskeletal system—has not been examined in a species‐specific morphofunctional context. Consequently, the morphofunctional basis underlying perching, climbing, inverted postures, and foot‐mediated food handling remains poorly understood.

Accordingly, the objectives of the present study were:

1. to describe and quantify the musculature responsible for digital flexion and extension in the red‐and‐green macaw;
2. to estimate digit‐specific gripping forces based on physiological cross‐sectional area (PCSA) and mechanical advantage; and
3. to elucidate the morphofunctional basis of complex foot‐use behaviors in this species, thereby highlighting the interplay among morphology, force production, behavior, and evolutionary history in shaping pedal prehension in macaws.

## 2 Materials and Methods

### 2.1 Specimens Studied and Dissection Procedures

The musculature responsible for digital flexion and extension was described, measured, and weighed in four adult specimens (three males and one female) of the red‐and‐green macaw (*Ara chloropterus* Gray, 1859). These specimens were obtained post mortem from individuals who had been accidentally electrocuted and subsequently recovered by the Arara Azul Institute (Campo Grande, Mato Grosso do Sul, Brazil). The frozen carcasses were donated to the Laboratory of Ecology, Systematics, and Conservation of Neotropical Birds (LESCAN/CPTL), where they were cataloged under accession numbers 77, 387, 405, and 625 and stored at −20°C. No animals were captured, handled, or manipulated in vivo for the purposes of this study. All procedures complied with Brazilian regulations governing the use of wildlife in scientific research. The transport of specimens was conducted under a permanent collection and transportation permit issued to Sérgio R. Posso by SISBIO/IBAMA (authorization 12221‐1).

Dissections of the hindlimbs were performed using a CENTAURO binocular stereomicroscope (10× eyepieces; 1.6× – 4× objectives) and standard dissection instruments, following the protocol of Previatto and Posso (2015). Muscles were exposed sequentially from superficial to deep layers, carefully detached from their origins and insertions, and identified. A 4% iodine solution was applied to enhance soft‐tissue contrast (Bock and Shear 1972).

The origin, insertion, and fiber orientation of each muscle were described and photographed using a 48 MP FHD digital camera (V8; Digilab) coupled to the stereomicroscope ocular to support anatomical documentation and illustration. After dissection, each muscle was weighed using a high‐precision semi‐analytical balance (Sartorius) (precision 0.001 g) to assess the proportional contribution of individual muscles. Mean muscle mass, along with its respective standard deviations, are presented in Table 1.

**Table 1:** Mean muscle length, mass, and physiological cross‐sectional area (PCSA) of the digital muscles in the red‐and‐green macaw, with corresponding standard deviations.

Muscle	Length (mm)	Mass (grams)	PCSA (mm^2^)
*M. flexor digitorum longus*	70.8 ± 5.7	0.7 ± 0.05	9.3 ± 3.3
*M. flexor hallucis longus*	84.8 ± 7.7	0.6 ± 0.05	9.6 ± 3.5
*M. flexor perforans et perforatus digiti II*	84.5 ± 7.1	0.3 ± 0.03	4.8 ± 2.0
*M. flexor perforans et perforatus digiti III*	60.8 ± 5.7	0.3 ± 0.04	3.4 ± 1.7
*M. flexor perforatus digiti II*	84.0 ± 6.9	0.88 ± 0.06	13.9 ± 4.1
*M. flexor perforatus digiti III*	63.50 ± 4.7	0.25 ± 0.02	3.0 ± 1.5
*M. flexor perforatus digiti IV*	84.8 ± 7.2	1.5 ± 0.02	24.0 ± 5.7
*M. flexor hallucis brevis*	33.2 ± 2.4	0.81 ± 0.05	5.1 ± 2.8

The specific morphological characteristics of each muscle—including origin, aponeurotic structures, fiber arrangement, and, most importantly, insertions on the respective phalanges of each digit—were documented in accordance with the *Nomina Anatomica Avium* (Baumel et al. 1993) and standardized anatomical nomenclature for muscle attachments.

### 2.2 Biomechanical Model

**Figure 1:**
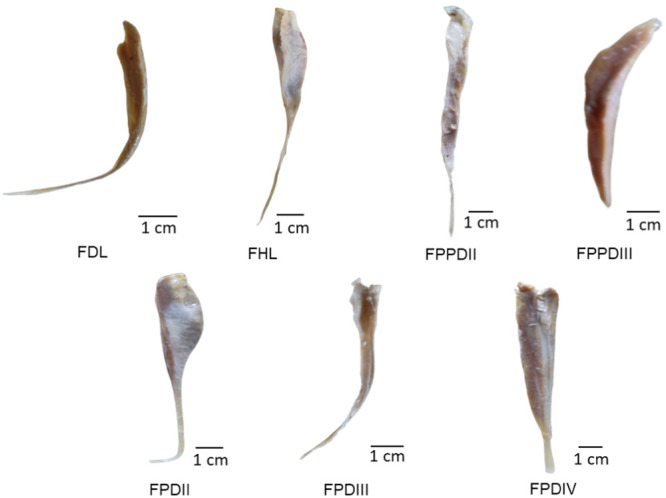
Images of the primary muscles involved in digital prehension in the red‐and‐green macaw. Legend: *m. flexor digitorum longus* (FDL), *m. flexor hallucis longus* (FHL), *m. flexor perforans et perforatus digiti II* (FPPDII), *m. flexor perforans et perforatus digiti III* (FPPDIII), *m. flexor perforatus digiti II* (FPDII), *m. flexor perforatus digiti III* (FPDIII), and *m. flexor perforatus digiti IV* (FPDIV).

To estimate muscle force‐generating capacity, we used the primary muscles involved in distal joint and digital prehension, i.e. *m. flexor digitorum longus* (FDL), *m. flexor hallucis longus* (FHL), *m. flexor perforans et perforatus digiti II* (FPPDII), *m. flexor perforans et perforatus digiti III* (FPPDIII), *m. flexor perforatus digiti II* (FPDII), *m. flexor perforatus digiti III* (FPDIII), *m. flexor perforatus digiti IV* (FPDIV), and *m. flexor hallucis brevis* (FHB) (Figures 1 and 3). These muscles are considered the primary contributors to distal joint flexion, digit rotation, and grasping performance during arboreal locomotion and food manipulation in birds (Baumel et al. 1993; Volkov 2004; Carril et al. 2014; Roderick et al. 2019; Dickinson et al. 2022).

Muscle forces associated with digital movements were initially calculated under isolated conditions following the explicit biomechanical framework of Sustaita (2008). Although the hindlimb was modeled independently to estimate muscular contributions to force output, osteological features relevant to the morphofunctional context were also considered when appropriate, following Bock (1964). These osteological observations were incorporated primarily in relation to the origins and insertions of the pedal flexor musculature, rather than as parameters of the mechanical model, in order to support functional interpretations.

**Figure 2:**
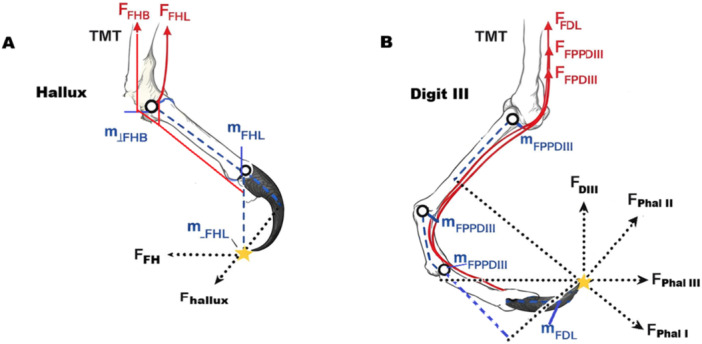
Biomechanical model of digital flexion in the Red‐and‐green Macaw (*Ara chloropterus*). Schematic representation of the lever systems operating in the digits, illustrating the relationship between muscle input force (F_in), output force (F_out), and their respective moment arms. (A) Digit I (hallux), (B) Digit III. Joint centers are indicated by black circles and correspond to the metatarsophalangeal and interphalangeal articulations, representing the centers of rotation used for moment calculations. Muscle input forces (F_in) are depicted as solid red arrows aligned with the line of action of the principal flexor tendons (e.g., *m. flexor digitorum longus*, *m. flexor hallucis longus*, *m. flexor perforans et perforatus digiti*, and *m. flexor perforatus digiti IV*), and are oriented in the direction of digital flexion. Output force (F_out) is represented by black dashed arrows originating at the distal tip of the ungual phalanx, corresponding to the reaction force generated during grasping or pinching. The in‐lever (m) is illustrated as solid blue lines and represents the perpendicular distance from the joint center to the line of action of F_in. The out‐lever (M) is illustrated as blue dashed lines and represents the distance from the joint center to the point of application of F_out at the ungual phalanx.

For mechanical advantage values, we considered each muscle acting about each phalangeal joint to obtain the ratio of the in‐lever moment arm [m] divided by that of the out‐lever [M]) (Figure 2). Thus, the mechanical advantage and digital flexion forces (Newtons) of the muscles acting on each digit (I–IV) were calculated. We also included the sum of the forces generated by each anterior and posterior digit as well as the total force of the feet in the red‐and‐green macaw (Table 2).

**Table 2:** Mechanical advantage and estimated digital pinching forces (Newtons) in the red‐and‐green macaw, with corresponding standard deviations.

Digit group	Digit	Mechanical advantage	Force (N)
Anterior digits	Digit III	0.130 ± 0.003	2.29 ± 0.3
	Digit II	0.140 ± 0.005	4.51 ± 0.92
	Summed		6.80 ± 1.22
Posterior digits	Digit I	0.154 ± 0.006	1.06 ± 0.15
	Digit IV	0.243 ± 0.013	8.63 ± 1.52
	Summed		9.69 ± 1.74
Total foot force			16.49 ± 3.22

The effective gripping force was then estimated by multiplying the summed muscular force by the mechanical advantage (MA) of the pedal lever system (Sustaita 2008). Mechanical advantage is defined as the ratio of the effective input lever arm to the output lever arm (Hildebrand and Goslow 2001).

Prior to muscle removal, we performed a series of biomechanical measurements to determine the input lever arms. Thus, input force lever arms (m) were measured in each specimen using a digital caliper, along the sagittal plane of flexion–extension, for each muscle acting on the digits. The mean line between the muscle origin and insertion was considered the line of action of the muscle, following Sustaita (2008). We defined the center of rotation at the midpoint of the articular contact between the tarsometatarsus and the proximal phalanges. Measurements were taken with the phalangeal joints positioned at 90°, which corresponds to the maximum moment‑arm length for these segments and approximates the joint angle. According to Sustaita (2008) food items were presumed to exert equal and opposite forces at this angle. Each moment arm was measured three times, and the mean was used for subsequent analyses.

For the calculation of the moment arms of the out‐forces, we measured the length from the tips of the claws to their extremity to consider the external lever arms of the nail‐phalangeal joints. Finally, we measured the lengths of the claw and phalangeal cords to obtain the remaining lengths of the external lever arms. All digit forces were modeled from the tips of the claws, which hypothetically constituted the initial points of food contact and the phalanges were treated as rigid bodies, following Sustaita (2008).

The mechanical advantage was calculated as: MA = in‐lever arm/out‐lever arm (Sustaita 2008). The mechanical advantage was estimated for two functional positions of the foot: the fully perching foot and the maximum observed foot opening in the red‐and‐green macaw. The maximum gape angle was determined by manually manipulating the fingers of all freshly collected specimens. In each specimen, the gape angle was measured directly using a protractor. These measurements yielded a mean maximum finger opening angle with a standard deviation.

### 2.3 Physiological Cross‐Sectional Area (PCSA) and Perching Force Estimation

Fascicle lengths and pennation angles were measured following the protocol of Sustaita (2008). After removal of the surrounding connective tissue while preserving internal muscle architecture, specimens were kept moist with 0.9% saline solution (NaCl) throughout the procedure to prevent dehydration. To facilitate fiber separation, muscles were immersed in 15% HNO_3_ for 24 h. Individual deep fibers were then carefully isolated and measured from their origin to insertion within the fascicle (excluding tendinous portions and overall muscle length). Fiber bundles (fascicles) were exposed along the longitudinal axis and to pennate muscles, representative regions of the muscle belly are selected because fiber length may vary within the muscle. Measurements were obtained from digital photographs and analyzed using ImageJ 1.30n (Schneider et al. 2012). For each muscle in each specimen, 10 fibers were sampled from ten different fascicles on both left and right sides. The mean fiber length calculated for each muscle with standard deviation was subsequently used to estimate PCSA following Sustaita (2008).

Pennation angles were measured relative to the longitudinal axis of the central tendon, with each muscle aligned so that the central tendon corresponded to the horizontal axis of a ruler. Mean pennation angles were subsequently calculated from calibrated digital photographs analyzed using ImageJ 1.30n software (Schneider et al. 2012). The angle measurements were then averaged for each individual muscle to calculate PCSA (Sustaita 2008).

PCSA was calculated by multiplying muscle mass by the cosine of the mean pennation angle and dividing by muscle tissue density (1060 kg m^−3^; Pennycuick 1996), followed by multiplication by mean fascicle length (Sustaita 2008). Finally, muscle force by PSCA for each muscle was estimated with a standard deviation (Table 1).

Finally, output force (F_out) was calculated following Hildebrand and Goslow (2001), using the equation F_out = F_in × (m/M), where *m* represents the in‐lever moment arm, *M* the out‐lever moment arm, and *F_in* the muscle force estimated from physiological cross‐sectional area (PCSA). We assume that the left and right perching muscles are symmetrical, and the latter percentage was calculated considering the sum of the measured muscles multiplied by two.

## 3 Results

### 3.1 Flexor Musculature Descriptions (Figures 1 and 3)

**Figure 3:**
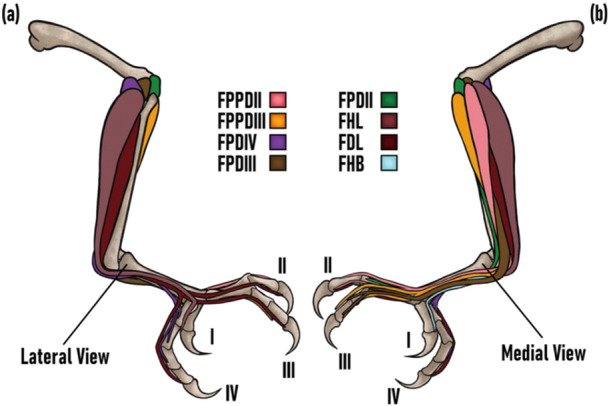
Primary muscles involved in digital prehension in the red‐and‐green macaw (*Ara chloropterus*), shown in lateral (left) and medial (right) views, illustrating their origins on the tibiotarsus and/or insertions on the respective phalanges. (a) lateral view of the hindlimb of the red‐and‐green macaw, (b) medial view of the hindlimb of the red‐and‐green macaw. Legend: *m. flexor digitorum longus* (FDL), *m. flexor hallucis longus* (FHL), *m. flexor perforans et perforatus digiti II* (FPPDII), *m. flexor perforans et perforatus digiti III* (FPPDIII), *m. flexor perforatus digiti II* (FPDII), *m. flexor perforatus digiti III* (FPDIII), and *m. flexor perforatus digiti IV* (FPDIV), and *m. flexor hallucis brevis* (FHB).

#### 3.1.1 M. Flexor Perforans et Perforatus Digiti II (FPPDII)

This is a bipennate muscle that originates by fleshy and tendinous fibers from the distal portion of the lateral femoral condyle and fibula. It extends distally along the lateral surface of the tibiotarsus, where it is caudally fused with the *m. flexor perforans et perforatus digiti III*. Its insertion occurs via a tendon that is perforated by the tendon of the *m. flexor digitorum longus*. Distally, the tendon courses along the plantar surface of the foot, crosses the first interphalangeal joint of digit II, and bifurcates to insert on both sides of the proximal end of the second phalanx, on its medial and lateral surfaces (Figure 3).

#### 3.1.2 M. Flexor Perforans et Perforatus Digiti III (FPPDIII)

This muscle has two bellies of origin on the lateral surface of the *tibiotarsus*, arising from the *crista cnemialis lateralis*, cranial to the *m. flexor perforans et perforatus digitorum*. It is a bipennate muscle that extends distally into a robust tendon. This tendon remains the most superficial as it courses along the plantar surface of the foot. Along its course, the insertion tendon is perforated by the tendon of the *m. flexor digitorum longus* and is connected to the tendon of the *m. flexor perforatus digiti III* by a flexor vinculum. Distally, the tendon bifurcates and inserts on both the lateral and medial surfaces of the base of the third phalanx of digit III (Figure 3).

#### 3.1.3 M. Flexor Perforatus Digiti IV (FPDIV)

This relatively large and elongate muscle lies deep to the *m. flexor perforans et perforatus digiti II* and *m. flexor perforans et perforatus digiti III*, and superficial to the *m. flexor perforatus digiti III*. Its main muscle belly occupies the caudolateral surface of the tibiotarsus. It originates from two bellies, both fleshy and tendinous, from the caudal surface of the distal region of the femur. These heads converge, and additional fibers arise from an associated aponeurosis, tapering distally into a broad, flattened tendon.

The tendon crosses the metatarsophalangeal joint and curves caudally to course along the plantar surface of digit IV. Four principal insertion sites are present on digit IV: (1) the midportion of the medial surface of the first phalanx; (2) the distal end of the medial surface of the second phalanx; (3) the lateral side of the proximal region of the third phalanx, where the tendon is perforated by the tendon of the *m. flexor digitorum longus*; and (4) the medial surface of the base of the fourth phalanx by the medial branch of this tendon (Figure 3).

#### 3.1.4 M. Flexor Perforatus Digiti III (FPDIII)

This muscle originates tendinously from the caudal surface of the distal end of the femur in the intercondylar region and presents two muscle bellies associated with a large aponeurosis. Distally, the two bellies converge, forming a broad tendon that is perforated by the tendons of the *m. flexor perforans et perforatus digiti III* and the *m. flexor digitorum longus*. The tendon then bifurcates distally, and the resulting lateral and medial branches insert on the lateral and medial surfaces, respectively, of the base of the second phalanx of digit III (Figure 3).

#### 3.1.5 M. Flexor Perforatus Digiti II (FPDII)

This muscle is well developed and originates fleshy from the caudal surface of the proximal portion of the fibula. It is positioned deep to the *m. flexor perforatus digiti III* along the caudal surface of the fibula and superficial to the *m. flexor hallucis longus*. The insertion tendon passes through the tibial cartilage and the hypotarsal groove, crosses the second metatarsophalangeal joint, and inserts on the lateral surface of the base of the first phalanx of digit II (Figure 3).

#### 3.1.6 M. Flexor Hallucis Longus (FHL)

This muscle is long and originates by fleshy and tendinous fibers from the caudal surface of the femur, proximal to the intercondylar area. It extends distally along the caudal surface of the tibiotarsus–fibula complex, lying deep to the *m. gastrocnemius*. As the fusiform muscle belly progresses distally, a dense aponeurosis develops, from which a strong, flat tendon emerges. This tendon passes through the hypotarsal canal, connects to the tendon of *m. flexor digitorum longus* via the *vinculum tendinum flexorum*, and inserts on the proximal end of the ventral surface of the ungual phalanx of digit I (Figure 3).

#### 3.1.7 M. Flexor Digitorum Longus (FDL)

It is a well‐developed, unipennate muscle with two bellies of origin, both fleshy and tendinous. The medial belly originates from the caudomedial surface of the tibiotarsus, whereas the lateral arises from the caudomedial surface of the fibula. Distally, the two bellies fuse and continue as a single insertion tendon, which passes through the hypotarsal canal and, in the distal region of the tarsometatarsus, connects to the tendon of *m. flexor hallucis longus* via a flexor vinculum. The tendon then divides into branches to insert on the flexor tubercle of each ungual phalanx and additionally on the distal end of phalanx III of digit III and phalanx IV of digit IV (Figure 3).

#### 3.1.8 M. Flexor Hallucis Brevis (FHB)

This is a short but relatively broad muscle located on the caudomedial surface of the tarsometatarsus, where it originates by two heads (proximal and distal), both fleshy and composed of robust fibers. The muscle lies deep to the digital flexor tendons. The broad and flattened tendon of the proximal head curves around the trochlea, crosses the metatarsophalangeal joint, and fuses with the thinner tendon of the distal head. The resulting tendon inserts on the lateral surface of the base of the first phalanx of digit I (Figure 3).

### 3.2 Flexor Musculature Measurements and Force Calculation

The length, mass and physiological cross‐sectional area (PCSA) of the muscles responsible for digital pinching are presented in Table 1.

All quantified muscles (Table 1) are involved in digital flexion and collectively contribute to the pinching action of the foot. Based on direct observations of muscle origins and insertions described above, we summarize below the muscles acting on each digit of the red‐and‐green macaw.

1. Anterior digits
  Digit II. Digital flexion is produced by the m. *flexor perforans et perforatus digiti II*, the m. *flexor perforatus digiti II*, and the m. *flexor digitorum longus*. Together, these muscles flex digit II primarily as a functional unit.
  Digit III. Flexion of digit III is generated by the m. *flexor perforans et perforatus digiti III*, the m. *flexor perforatus digiti III*, and the m. *flexor digitorum longus*, which likewise act to flex the digit predominantly as a unit.
2. Posterior digits
  Digit I (hallux). Flexion of the hallux involves two muscles with complementary roles. The m. *flexor hallucis brevis* flexes the digit as a unit, whereas the m. *flexor hallucis longus* is recruited when flexion of individual phalanges is required.
  Digit IV. Flexion of digit IV is produced mainly by the m. *flexor perforatus digiti IV*, which acts both in flexing individual phalanges and in flexing the digit as a unit. Additional contributions are provided by the m. *flexor digitorum longus* and the m. *flexor hallucis longus*, which also participate in flexion of the digit as a whole.

Thus, by summing the estimated forces generated by the muscles acting on each digit, we calculated the mechanical advantage and total force exerted by digits I–IV individually, the combined digital pinching force of the anterior and posterior digits in the zygodactyl foot, and the overall pinching force of the foot (Table 2).

### 3.3 Extensor Musculature Descriptions (Figure 4)

**Figure 4:**
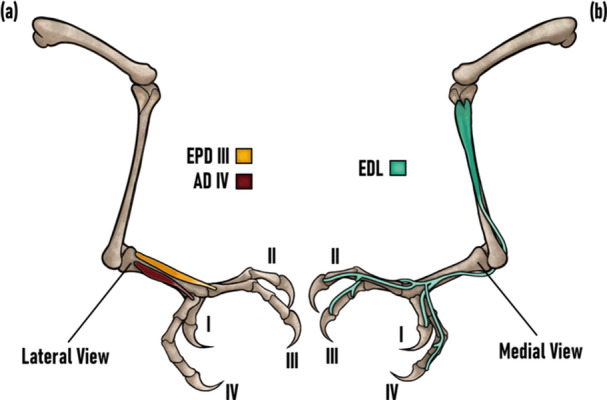
Primary muscles involved in digital extension in the red‐and‐green macaw (*Ara chloropterus*), shown in lateral (left) and medial (right) views, illustrating their origins on the metatarsus and tibiotarsus and/or insertions on the respective phalanges. (a) lateral view of the hindlimb of the red‐and‐green macaw, (b) medial view of the hindlimb of the red‐and‐green macaw. Legend: *m. abductor digiti IV* (ADIV), *m*. *extensor digitorum longus* (EDL), *m. extensor proprius digiti III* (EPDIII).

#### 3.3.1 *M. abductor digiti IV* (ADIV, Figure 4)

The *m. abductor digiti IV* is a small intrinsic pedal muscle originating from the ventrolateral surface of the proximal tarsometatarsus. Its slender tendon extends distally to insert on the base of the proximal phalanx of digit IV. The muscle is markedly reduced and lies medially to the tendinous portion of the *m. gastrocnemius*.

#### 3.3.2 *M. extensor proprius digiti III* (EPDIII, Figure 4)

The *m. extensor proprius digiti III* is a slender and elongated muscle originating from the dorsal surface of the tarsometatarsus, deep to the tendon of the *m. extensor digitorum longus*. Its tendon courses distally along the dorsal aspect of digit III and inserts on the dorsal surface of the proximal phalanx.

#### 3.3.3 *M. extensor digitorum longus* (EDL, Figure 4)

The *m. extensor digitorum longus* is slender and pinnate, this muscle lies along the anteromedial surface of the tibiotarsus. It is an extrinsic extensor of the pes. The origin is fleshy from most of the region between the cnemial crests, within the *sulcus intercnemius*. Distally, the muscle forms a robust tendon that divides into two terminal branches supplying digits I–IV. The first branch is the tendon to digit I (hallux), and it inserts on the extensor tubercle of the ungual phalanx. The tendon further subdivides into two branches. The first one supplies the digit IV, inserting on the extensor tubercle of the ungual phalanx and on the base of the middle phalanx. The second branch supplies the digits II and III. In digit II, the tendon inserts proximally on the medial and lateral aspects of the base of the proximal phalanx and distally on the extensor tubercle of the ungual phalanx. In digit III, the tendon bifurcates, inserting on the extensor tubercle of the ungual phalanx, the dorsal surface of the distal end of the proximal phalanx, and the dorsal‐lateral surface of the middle phalanx.

The following muscles are absent in the Red‐and‐green Macaw: (1) *m. adductor digiti II*; (2) *m. adductor digiti IV*; *m. abductor digiti II; M. extensor hallucis longus*; *m. extensor brevis digiti III; m. extensor proprius digiti IV; m. extensor brevis digiti IV; m. lumbricalis* and *m. plantaris*.

## 4 Discussion

Initial anatomical investigations of Psittaciformes were based on early descriptive works that provide general overviews for a limited number of species (Lakjer 1926; Lubosch 1933; Moller 1950; Dubale and Rawal 1965). Most recently, most studies focused primarily on cranial anatomy (Hofer 1950, 1953; Dubale and Rawal 1965; Burton 1974; Porto 2004; Tokita 2004, 2006; Tokita et al. 2013; Carril et al. 2015; Faillace et al. 2025; Posso et al. 2026), whereas investigations of the hindlimb have been notably limited (Sustaita et al. 2013; Dickinson et al. 2022). To date, detailed anatomical study of the hindlimb in psittacines has been limited to Carril et al.'s (2014) description of the Monk Parakeet (*Myiopsitta monachus*). Additionally, Roderick et al. (2019) and Dickinson et al. (2022) performed an in vivo measurement of grip force in the Pacific Parrotlet (*Forpus coelestis*) and Rosy‐faced Lovebird (*Agapornis roseicollis*), respectively. Comparable analyses have not previously been conducted in macaws and studies involving detailed measurements and descriptions of macaws' musculature remain relatively scarce (Posso et al. 2026).

### 4.1 Functional Significance and Evolutionary Implications of the Zygodactyl Foot: Muscular Adaptations for Grasping and Manipulation

The ancestral avian pedal morphology is anisodactyl (Bock and Miller 1959), characterized by a posteriorly directed hallux (digit I) and anteriorly oriented digits II, III, and IV (Feduccia 1999; Carril et al. 2024). Recent phylogenetic studies based on molecular data (Hackett et al. 2008; Jarvis et al. 2014; Prum et al. 2015; Kimball et al. 2019; McTavish et al. 2025) support a close evolutionary relationship between Psittaciformes and Passeriformes, which together form the clade Psittacopasserae (Suh et al. 2011). While Passeriformes retain the general anisodactyl arrangement, digit IV is often positioned more laterally than digits II and III and may exhibit limited abduction (Botelho et al. 2014). This condition, however, does not constitute true ectropodactyly—as observed in woodpeckers (Picidae)—because digit IV remains primarily anteriorly oriented (Carril et al. 2024). In passerines, which are generally small and lightweight, the hallux alone provides sufficient grasping capability for perching.

Nevertheless, molecular phylogenetic and paleontological evidence suggests that the passerine ancestor may have been zygodactyl, with a subsequent anterior shift of digit IV during evolutionary history (Botelho et al. 2014). If this reconstruction is accurate, the ancestral psittacopasseran likely exhibited a fully reversed digit IV, positioned posteriorly alongside the hallux and resulting in a functional zygodactyl foot (Carril et al. 2024).

Under this interpretation, Psittacinae retain a plesiomorphic zygodactyl condition, while secondarily evolving an elongated digit IV and a particularly large and robust *m. flexor perforatus digiti IV*. This configuration is commonly interpreted as an adaptation to perching and arboreal locomotion in a lineage that also relies heavily on the feet for vertical positioning and food manipulation (Collar 1997; Backus et al. 2015). Ontogenetic analyses of the budgerigar (*Melopsittacus undulatus*) by Botelho et al. (2014) indicate that the reversal of digit IV is associated with asymmetries in pedal musculature, particularly the reduction of the *m. extensor digiti IV brevis*. From this perspective, epigenetic factors and patterns of muscle activity during development may play a primary role in shaping the zygodactyl foot, rather than a strictly phylogenetic explanation for the diversification of pedal configurations within Psittacinae (Carril et al. 2021). These interpretations suggest that zygodactyl feet in Psittaciformes represent distinct adaptive solutions for enhancing grasping performance, including increased contact area and more symmetrical force distribution among digits during substrate or feeding interaction.

Although the study of pedal myology in *Myiopsitta monachus* by Carril et al. (2015) did not include force estimations, digit IV in that species is likewise four elongated phalanges and associated with robust musculature involved in its movement. This robustness of digit IV is more pronounced in the Red‐and‐green Macaw than in *M. monachus*, consistent with the substantially larger body size of this macaw. As demonstrated here, the zygodactyl condition in the red‐and‐green macaw is accompanied by the development of robust digital flexor musculature, particularly the enlarged *m. flexor perforatus digiti IV*, associated with the elongated digit IV. This muscle contributes substantially to the high force output of digit IV, which exceeds that of all other digits. Because the hallux is relatively smaller and generates the lowest grip force, the well‐developed digit IV compensates by contributing disproportionately to posterior force production. In fact, this pattern is convergent and common among tetrapods and follows rules of skeletal development (Abourachid et al. 2017). As a result, force distribution between anterior and posterior digits is more evenly balanced, enhancing mechanical stability and load sharing during arboreal locomotion, particularly in large‐bodied birds such as Red‐and‐green Macaws. This arrangement enables effective perching (Bock and Miller 1959) on substrates of varying diameter and orientation, reducing energetic costs associated with postural maintenance and minimizing the risk of slipping or loss of balance (Roderick et al. 2019). In addition, this pedal configuration enables climbing, hanging, maintenance of inverted postures during foraging, and controlled terrestrial locomotion (Collar 1997). These peculiar aspects of arboreal locomotion and inverted foraging behavior are well illustrated by photographic documentation of red‐and‐green macaws in Scherer‐Neto and Terto (2011).

The zygodactyl feet function not only as locomotor structures but also as manipulative organs that play a central role in feeding behavior (Collar 1997). The *mm. flexores perforantes et perforati digitorum II et III* are caudally fused in the Red‐and‐green Macaw and in *Amazona albifrons* (Berman 1984). This muscular integration may facilitate a pincer‐like action involving digits I, II, and III, thereby enhancing the fine motor ability to grasp and manipulate larger and heavier food items. Macaws routinely use the hindlimbs to grasp and stabilize large food items, transferring them to the beak while maintaining postural equilibrium on unstable substrates (Collar 1997; Backus et al. 2015). During foraging, individuals frequently anchor one or both feet to branches, allowing the body to rotate into head‐down or inverted postures (Scherer‐Neto and Terto 2011; Backus et al. 2015). Such behaviors require precise coordination between digital flexor musculature and joint stabilization mechanisms (Carril et al. 2014, 2024). Another indication of adaptation for fine motor manipulation is the well‐developed *m. extensor digiti III brevis* in the Red‐and‐green‐Macaw, which exhibits an extensive area of origin and likely contributes to enhanced independent control and positional adjustment of digit III.

Psittacids routinely manipulate food items with the feet, and zygodactyly has long been interpreted as an adaptation enhancing grasping efficiency by producing two opposing pairs of digits (Bock and Miller 1959; Roderick et al. 2019; Dickinson et al. 2022; Carill et al. 2024). In this context, the extensor and intrinsic musculature of the pes appears to play a crucial role in digital coordination and stabilization.

The intrinsic musculature associated with digit abduction and stabilization clearly reflects arboreal specialization in parrots. As an example, the *m. abductor digiti IV* is an intrinsic hindlimb muscle responsible for the abduction and lateral orientation of the fourth toe. It originates on the tarsometatarsus and is crucial for foot dexterity, playing a key role in grasping and climbing (Roderick et al. 2019; Dickinson et al. 2022). This muscle is present in most avian groups examined, including parrots, raptors, and ratites (Bock and Miller 1959; Roderick et al. 2019; Dickinson et al. 2022; Carill et al. 2024), but its functional role differs according to pedal configuration (Berman 1984; Picasso 2010). In anisodactyl birds, this muscle primarily acts as an abductor of digit IV, whereas in zygodactyl taxa its altered orientation likely causes it to function biomechanically as an extensor of the retroverted fourth digit (Berman 1984; Dickinson et al. 2022; Carill et al. 2024). Such a functional shift may contribute to stabilization of the posterior digits in the red‐and‐green macaw during grasping and climbing.

The *m. flexor hallucis brevis* is a crucial superficial flexor muscle in the bird's hindlimb that independently flexes the hallux (Baumel et al. 1993). It is highly specialized for fine‐tuned toe positioning and plays a key mechanical role in perching and grasping in the red‐and‐green macaw. Thus, its anatomical arrangement suggests a role in stabilization and controlled flexion of the hallux, contributing to oppositional grasping in the zygodactyl foot (Dickinson et al. 2022; Carill et al. 2024). The *m. flexor hallucis brevis* is comparatively less developed in macaws than in raptors (Sustaita 2008), probably because macaws frequently rest within cavities (Forshaw 1989; Collar 1997) rather than relying exclusively on prolonged perching. Raptors and passerines that habitually sleep perched require strong hallux flexion to maintain digital locking during extended periods of inactivity (Kaiser 2007).

The extensor musculature of parrots also exhibits modifications associated with refined digital control (Berman 1984; Dickinson et al. 2022; Carill et al. 2024). The *m. extensor proprius digiti III* is located on the dorsal (anterior) surface of the tarsometatarsus and its primary role is to straighten or extend the third digit (toe III). Thus, this muscle likely functions in independent extension and positional adjustment of digit III, enhancing digital coordination during perching and manipulative behaviors. According to George and Berger (1966) and Vanden Berge and Zweers (1993), the *m. extensor proprius digiti III* is not a consistently developed muscle across birds. Instead, it is one of the most phylogenetically variable intrinsic muscles of the avian pes, ranging from complete absence to a well‐developed, functionally specialized muscle in taxa that exhibit complex pedal dexterity. This extensor muscle is reduced or absent in ratites (Hudson et al. 1972) and many raptorial birds (Mosto et al. 2013) and highly modified in Psittaciformes (Berman 1984). Most probably, the enlargement and differentiation of this muscle observed here in the Red‐and‐green Macaw, as well as in other parrots, are adaptations for increased independence of digit III during grasping and object manipulation, complementing the specialized zygodactyl configuration of the foot rather than contributing substantially to locomotor performance. Likewise, the highly subdivided distal tendon system of the *m. extensor digitorum longus* indicates an advanced degree of digital coordination, allowing simultaneous stabilization and repositioning of all digits during grasping, climbing, and grasp release. In combination with antagonistic flexor systems, this muscle forms part of the principal neuromuscular network controlling pedal prehension in parrots (Berman 1984). Such elaboration of the extensor apparatus contrasts with the condition in raptorial birds, where functional specialization primarily emphasizes flexor hypertrophy and forceful prey restraint rather than refined manipulative control (Ward et al. 2002; Sustaita 2008).

The *m. extensor proprius digiti III* together with the accessory branch of the *m. extensor digitorum longus* to the hallux while the *m. extensor hallucis longus* is markedly reduced (Berman 1984), illustrating the evolutionary enhancement of intrinsic pedal control associated with arboreal locomotion, climbing, and exceptional manipulative abilities of parrots and their zygodactyl foot. A nearly identical arrangement occurs in mousebirds (Coliiformes) (Berman and Raikow 1982), suggesting that coordinated control of all four digits may facilitate the complex pedal behaviors shared by both groups, including climbing, inverted postures, and object manipulation. Despite these morphological similarities, Psittaciformes and Coliiformes are not sister taxa (McTavish et al. 2025). Consequently, the shared pedal muscular specializations are most likely the product of convergent evolution associated with similar functional demands, rather than retention of homologous features from a common ancestor.

The Red‐and‐green Macaw exhibits this same derived configuration, with the *m. extensor digitorum longus* replacing the function of the absent *m. extensor hallucis longus* by supplying an independent tendon to the hallux. Such an arrangement likely enhances synchronization among all four digits during extension while maintaining precise positional control of the opposable hallux. Rather than acting solely to extend the digits during locomotion, the *m. extensor digitorum longus* in parrots appears to function as a major coordinator of digital repositioning during grasp release, climbing, inverted suspension, and food manipulation (Berman 1984). Its action, antagonistic to the powerful *m. flexor digitorum longus* and *m. flexor hallucis longus*, forms the principal mechanical system responsible for regulating transitions between forceful grasping and controlled release (Vanden Berge and Zweers 1993). This interpretation is consistent with recent evolutionary analyses demonstrating that, whereas the *m. flexor digitorum longus* constitutes one of the most conserved extrinsic muscles of the avian pes (Carril et al. 2024), modifications of its distal tendon arrangement have played a pivotal role in the evolution of grasping and arboreal behaviors by increasing the functional integration and independence of the digits in specialized taxa such as parrots.

Variation in intrinsic musculature among zygodactyl birds may also provide important insights into the developmental and evolutionary origins of digit retroversion. In particular, the presence, reduction, or absence of the *m. extensor digiti IV brevis* may be directly related to the ontogenetic establishment of zygodactyly. Developmental analyses by Botelho et al. (2014) demonstrated that retroversion of digit IV in parrots is associated with asymmetrical muscular development during ontogeny, including modifications of intrinsic extensor musculature. These findings suggest that zygodactyly may arise not only from skeletal rearrangement, but also through epigenetic interactions involving muscular imbalance and altered tendon mechanics during development (Botelho et al. 2014). Consequently, comparative analyses of intrinsic pedal musculature among parrots, woodpeckers, cuckoos, owls, and collies may help clarify whether superficially similar zygodactyl conditions evolved through homologous developmental pathways or represent convergent biomechanical solutions to similar functional demands.

The extensive tendon branching and multiple insertion sites indicate that the *m. extensor digitorum longus* plays a major role in coordinated digital extension, stabilization, and repositioning of the zygodactyl foot during locomotion, climbing, and grasp release (Dickinson et al. 2022; Carill et al. 2024). In the Red‐and‐green Macaw this muscle performs the function of the *m. extensor hallucis longus*, which is absent in this macaw. In combination with antagonistic flexor muscles, the *m. extensor digitorum longus* is essential for unlocking and releasing the grip on a branch when the bird takes off from a perch (Dickinson et al. 2022; Carill et al. 2024). Along with the *m. flexor digitorum longus*, these antagonistic flexor muscles form the core mechanical network that enables fine motor control of the digits. The branch of the *m. extensor digitorum longus* supplying the hallux, observed in the Red‐and‐green Macaw, has also been described in other parrots and in *Colius striatus* (Berman and Raikow 1982), a pamprodactyl bird capable of complex pedal manipulation and inverted postures. It is possible that the ability to maintain inverted (descending) postures is enhanced in both avian orders by the condition in which all four digits are coordinated through a single extensor muscle system. In anisodactyl birds, independent control of the hallux is generally maintained through separation from the anterior digits, because digit I alone acts in opposition during perching and it is enlarged in climbing species (Abourachid et al. 2017). In parrots and collies, however, the integration of all four digits through a common extensor system may facilitate coordinated manipulative movements and enhanced pedal dexterity (Berman 1984). In the Red‐and‐green Macaw, the absence of the *m. extensor hallucis longus* is functionally compensated by the hallux branch of the *m. extensor digitorum longus*, a condition also described in *Amazona albifrons* (Berman 1984).

Other muscular specializations may relate directly to grasping mechanics. The insertion of the *m. ambiens* onto the tendon of the *m. flexor perforatus digiti IV* in macaws resembles the condition described for Cathartidae and differs from the insertions observed in ratites (Fisher 1946; Picasso 2010). This arrangement likely contributes to automatic digital flexion during intertarsal joint flexion, facilitating secure grasping of branches during perching (Kaiser 2007). Likewise, fusion of the *mm. flexores perforantes et perforati digitorum II et III*, observed in the Red‐and‐green Macaw and in *Amazona albifrons* (Berman 1984), may enhance coordinated pinching movements among digits I–III, potentially improving manipulation of large and heavy food items. Such muscular integration is especially relevant in macaws, whose generalized diet includes large fruits, seeds, and other resistant plant materials requiring complex pedal manipulation (Forshaw 1989; Collar 1997).

Together, these observations indicate that the hindlimb musculature of macaws combines the generalized avian muscular plan with a series of quantitative and qualitative modifications associated with arboreal locomotion, climbing, and manipulative prehension. The hindlimb musculature of the macaws examined here was broadly similar to that described for other parrots, particularly with respect to the origins and insertions of the different muscle groups previously documented in *Amazona albifrons* (Berman 1984) and *Monachus monachus* (Carril et al. 2014). Thus, the elaboration of intrinsic and extensor musculature, coupled with modifications of tendon architecture and digital coordination, appears to have been fundamental to the evolution of the highly dexterous zygodactyl foot characteristic of Psittaciformes.

### 4.2 Pedal Muscular Morphology and Grip Force Production

Dickinson et al. (2022) measured pedal prehensile force in the Rosy‐faced Lovebird (*Agapornis roseicollis*), reporting a maximum force of approximately 4 N, which decreased with increasing perch diameter—consistent with pedal morphology specialized for grasping slender substrates. A comparable pattern was reported for the Pacific Parrotlet (*Forpus coelestis*) by Roderick et al. (2019). Although the maximum forces recorded in these small parrots are approximately one‐quarter of those measured in the red‐and‐green macaw, this disparity is best interpreted in the context of their markedly smaller body size (~15 cm total length, 40–50 g; Collar 1997). The substantially greater absolute assisted grip strength observed in the Red‐and‐green Macaw likely reflects its markedly larger body mass—exceeding that of small psittacines by more than 30‐fold—which presumably necessitates enhanced pedal force production to maintain postural stability, particularly during descending movements or inverted foraging postures. Behavioral study in parrots further indicates that foot use in feeding is associated with lateralization and body size, with larger species exhibiting stronger limb musculature (Brown and Magat 2011). However, future studies should extend pedal grip‐force measurements across a broader taxonomic range of Psittaciformes and apply formal allometric analyses to test scaling relationships between body size and pedal force, following approaches analogous to those used by Harrison et al. (2024) in analyses of bite‐force scaling in parrots.

Comparisons with raptorial taxa reveal marked functional divergence in pedal performance. In hawks and falcons, the pes is specialized for prey capture, subjugation, and killing, with performance driven by hypertrophied digital flexor musculature and high mechanical advantage that maximize forceful grasping (Sustaita 2008). In contrast, parrots and collies emphasize dexterity and fine motor control, supported by increased differentiation of intrinsic musculature and specialized tendon insertions that enhance digital independence and precision (Berman and Raikow 1982; Berman 1984). Our results for the red‐and‐green macaw are consistent with this pattern. This macaw exhibits lower overall and digit‐specific force outputs, reflecting divergent functional demands. Rather than maximizing mechanical power, the macaw pes is characterized by refined muscular coordination and enhanced dexterity, features essential for precise food manipulation, controlled arboreal locomotion, inverted postures, and climbing behaviors (Collar 1997; Backus et al. 2015). Nevertheless, some avian taxa, including certain accipitrids and falconids, exhibit notable abilities in object manipulation with the feet (Burton 1978; Biondi et al. 2008), suggesting that prehensile performance extends beyond force production alone.

Consistent with this interpretation, marked differences are evident when comparing individual digit forces between Red‐and‐green Macaws and Falconiformes and Accipitriformes. In these raptors, the hallux is typically among the strongest digits, reflecting its central role in prey capture (Sustaita 2008). In the red‐and‐green macaw, however, the hallux generated relatively low force (1.06 N; Table 2). This reduced contribution is compensated by the markedly greater force produced by digit IV, which exceeds hallux force values reported for several falcon and hawk species, including *Falco sparverius* (1.25 N), *Falco columbarius* (1.22 N), *Accipiter striatus* (0.92 N), and *Accipiter cooperii* (3.69 N), and approaches or exceeds values observed in larger falcons (Sustaita 2008).

Digit II in the red‐and‐green macaw also exhibited relatively high force (4.51 N), exceeding that of the hallux and surpassing values reported for some falcon species (Sustaita 2008). In contrast, digit III displayed the lowest force between the anterior digits, a pattern that parallels findings in raptors, where digit III similarly contributes less to overall force production (Sustaita 2008). However, the total foot force in red‐and‐green macaw (16.49 N) only exceeded values reported for several smaller falcons and hawks, such as *Falco sparverius* (6.68 N), *Falco columbarius* (7.99 N), and *Accipiter striatus* (5.34 N), but remained lower than that observed in larger raptors, including *F. mexicanus* (27.19 N) and *Falco peregrinus* (25.69 N) (Sustaita 2008). Only the value for *A. cooperii* (16.81 N, Sustaita 2008) was comparable to that of the Red‐and‐green Macaw. These comparisons should be interpreted in light of body mass differences: the Red‐and‐green Macaw is substantially heavier (1250–1700 g; Guedes 2012; Sick 2001) than most falcons and hawks examined by Sustaita (2008), often by a factor of three to five. Thus, rather than being optimized for prey capture, the digital musculature of the red‐and‐green macaw is adapted to an arboreal lifestyle, in which considerable prehensile force is required to support large body weight, move efficiently among branches, and maintain inverted postures during foraging (Collar 1997). However, these functions depend not only on the digital flexors examined here, but also on other components of the hindlimb musculature, which warrant further investigation to fully understand pedal kinetics in macaws. Collectively, these features underscore the functional integration of pedal morphology, musculature, and behavior in macaws, reflecting strong selective pressures associated with an arboreal lifestyle, large body size and complex foot manipulative behavior.

Finally, the detailed characterization of the musculature responsible for digital movements performed here provides a critical framework for comparative anatomy, anatomical atlases, systematics, veterinary applications, functional paleontology, and evolutionary analyses in macaws. In addition, when integrated with biomechanical and performance‐based approaches, these data refine our understanding of the structural and functional diversity of the macaw hindlimb. This integrative approach is essential not only for elucidating functional mechanisms, but also for informing broader evolutionary interpretations of pedal grasping systems across birds, thereby enabling the identification of lineage‐specific specializations associated with locomotion, manipulation, and behavioral ecology. Further comparative analyses integrating developmental biology, functional morphology, and biomechanical modeling will be essential for clarifying the evolutionary pathways underlying pedal grasping systems in birds.

## 5 Conclusions

The elongated digit IV, powered by the largest and heaviest digital flexor (*m. flexor perforatus digiti IV*), generates the greatest individual digit force in the red‐and‐green macaw. This powerful digit IV promotes force distribution between anterior and posterior digits. This near‐symmetry provides mechanical stability during perching and arboreal locomotion, a critical adaptation for a large‐bodied and arboreal manipulator bird. The robust feet musculature and high force output are consistent with the macaw's large body size, fulfilling the biomechanical demands of weight support and dynamic postures. The study provides foundational data supporting the hypothesis that pedal force scales with body size in Psittaciformes, a relationship requiring formal allometric testing across more species. While the macaw's total pedal force is substantial, it is lower than that of similarly sized raptors specialized for prey capture. This indicates an evolutionary trade‐off where the foot is optimized for prehensile dexterity, controlled climbing, and inverted postures rather than maximizing crushing power—a role fulfilled in macaws by the beak.

## Author Contributions

**Sérgio Roberto Posso:** conceptualization, project administration, methodology, data curation, investigation, validation, formal analysis, writing – original draft, writing – review and editing and supervision. **Vinicius Camargo Tavares:** conceptualization, methodology, data curation, investigation, validation, formal analysis, writing – original draft. **Ana Caroline M. Barbosa:** visualization, writing – review and editing.

## Funding

The authors have nothing to report.

## Ethics Statement

This study was based exclusively on specimens cataloged in collections, and no live animals were collected or experimentally manipulated.

## Conflicts of Interest

The authors declare no conflicts of interest.

## Data Availability

The data that support the findings of this study are available on request from the corresponding author. The data are not publicly available due to privacy or ethical restrictions. All data supporting the findings of this study are included within the manuscript. No additional datasets were generated or analyzed.
